# Myogenesis in the basal bilaterian *Symsagittifera roscoffensis *(Acoela)

**DOI:** 10.1186/1742-9994-5-14

**Published:** 2008-09-19

**Authors:** Henrike Semmler, Xavier Bailly, Andreas Wanninger

**Affiliations:** 1 University of Copenhagen, Department of Biology, Research Group for Comparative Zoology, Universitetsparken 15, DK-2100 Copenhagen Ø, Denmark; 2 Station Station Biologique de Roscoff, Place Georges Teissier BP74, F-29682 Roscoff Cedex, France

## Abstract

**Background:**

In order to increase the weak database concerning the organogenesis of Acoela – a clade regarded by many as the earliest extant offshoot of Bilateria and thus of particular interest for studies concerning the evolution of animal bodyplans – we analyzed the development of the musculature of *Symsagittifera roscoffensis *using F-actin labelling, confocal laserscanning microscopy, and 3D reconstruction software.

**Results:**

At 40% of development between egg deposition and hatching short subepidermal fibres form. Muscle fibre development in the anterior body half precedes myogenesis in the posterior half. At 42% of development a grid of outer circular and inner longitudinal muscles is present in the bodywall. New circular muscles either branch off from present fibres or form adjacent to existing ones. The number of circular muscles is higher than that of the longitudinal muscles throughout all life cycle stages. Diagonal, circular and longitudinal muscles are initially rare but their number increases with time. The ventral side bears U-shaped muscles around the mouth, which in addition is surrounded by a sphincter muscle. With the exception of the region of the statocyst, dorsoventral muscles are present along the entire body of juveniles and adults, while adults additionally exhibit radially oriented internal muscles in the anterior tip. Outer diagonal muscles are present at the dorsal anterior tip of the adult. In adult animals, the male gonopore with its associated sexual organs expresses distinct muscles. No specific statocyst muscles were found. The muscle mantles of the needle-shaped sagittocysts are situated along the lateral edges of the animal and in the posterior end close to the male gonopore. In both juveniles and adults, non-muscular filaments, which stain positively for F-actin, are associated with certain sensory cells outside the bodywall musculature.

**Conclusion:**

Compared to the myoanatomy of other acoel taxa, *Symsagittifera roscoffensis *shows a very complex musculature. Although data on presumably basal acoel clades are still scarce, the information currently available suggests an elaborated musculature with longitudinal, circular and U-shaped muscles as being part of the ancestral acoel bodyplan, thus increasing the possibility that Urbilateria likewise had a relatively complicated muscular ground pattern.

## Background

Acoela, supposedly the earliest recent bilaterian offshoot, show only few morphological characters to infer their evolution and their phylogenetic relationships [1]. They are acoelomate, gutless ciliated worms mainly from marine habitats and have traditionally been assigned to the taxon Turbellaria within Platyhelminthes [2]. At present, three major flatworm-like clades are often considered valid, namely Platyhelminthes, Nemertodematida and Acoela, of which the latter two often are considered sistergroups and combined in the phylum Acoelomorpha [3-5], although other hypotheses, that argue for Acoela and Nemertodermatida as two distinct phyla, do exist [1,6-8]. Within the Acoela, the interrelationship of taxa still remains unclear due to the lack of morphological characters [9]. A recent molecular survey using the 18S rDNA gene as a marker also supports the hypothesis that most current acoel families are polyphyletic [10]. Morphological characters used to infer acoel interrelationships focus mainly on the male genital organs, but female genital organs, the structure of the epithelium, the nervous system, the pharynx and the bodywall musculature are also included in these analyses [11].

The architecture of the musculature has been proven useful to infer higher sistergroup relationships within, e.g., *Childia *and *Convoluta*, where specific muscle patterns as well as sperm and stylet ultrastructure challenge their monophyly [10,12,13]. Recently, the phylogeny of Convolutidae *sensu *[11] underwent some revisions, in which all small Convolutida clustered together and are now classified in the newly erected taxon Isodiametridae outside the Convolutidae, based on their penis musculature [14]. Other analyses suggest that all taxa that possess sagittocysts (i.e., needle-shaped secretory products), e.g., *Symsagittifera roscoffensis*, cluster in the monophyletic assemblage Sagittiferidae [15,16].

In earlier studies the arrangement of the bodywall musculature was mainly characterized as part of the species description without using it as a character for evolutionary inferences e.g., [11,17-19]. However, recent studies showed that variations in the muscular arrangement may be successfully used for phylogenetic analysis [12]. Differences in shape, number, thickness, orientation, arrangement and presence/absence of muscles have been proven useful [20], and descriptions of the adult musculature, mainly of the bodywall, were carried out on more than 50 acoel species by applying fluorescence-coupled phalloidin e.g., [12,21-26]. As such, the adult musculature was also investigated in the three sagittiferid species *Antrosagittifera corallina*, *Convolutriloba longifissura *and *Convolutriloba macropyga *[12,24,26]. With the present study we add to the scarce data on acoel muscle formation by providing detailed 3D reconstructions of the muscular architecture of various stages of *Symsagittifera roscoffensis *(Graff, 1891), which is currently also the subject of an extensive EST programme. We compare our data to the data available on adult acoel muscle bodyplans as well as to the sole study on acoel myogenesis available to date on *Isodiametra pulchra *[27].

## Methods

### Animals

Adults were collected in the upper part of the intertidal zone on the Brittany Coast on the Ile Calot close to Carantec, France. Adults were kept in aquaria with natural, unfiltered seawater at the Station Biologique de Roscoff until egg laying was observed. Cocoons containing embryos were isolated and cultured in sea water.

Prior to fixation, 7% MgCl_2 _was applied to avoid muscle contraction. The specimens were fixed in 4% paraformaldehyde in 0.1 M phosphate buffer (PB) for 0.5–2 hours at room temperature. The specimens were washed several times in PB and stored at 4°C in 0.1 M PB with 0.1% sodium azide (NaN_3_) added to prevent bacterial or fungal contamination.

On the first day of egg deposition several cocoons with 2, 4, 8, 16, 32 and 64 cell stages were fixed, on the second day embryos were fixed thrice, and from the third day onwards embryos and juveniles were fixed once daily.

### F-actin staining, data acquisition and 3D reconstruction

For F-actin visualization the specimens were incubated in 1:40 diluted Alexa Fluor 488 phalloidin (Molecular Probes, Eugene, OR, USA) in 0.1 M PB containing 1% Triton X-100 and 0.1% NaN_3_. After the overnight incubation three washes in 0.1 M PB followed. The specimens were mounted on Poly-L-lysine coated cover slips in Fluoromount-G (Southern Biotech, Birmingham, AL, USA) and stored at 4°C.

About 10 specimens per stage were analysed with a Leica DM IRE 2 fluorescence microscope with a TCS SP2 AOBS confocal laserscanning device (Leica Microsystems, Wetzlar, Germany). Images were edited with the Leica confocal software, Adobe Photoshop CS2 and Adobe Illustrator CS2 imaging softwares, respectively (Adobe Systems; San Jose, CA, USA). For 3D reconstruction, the CLSM image stacks were processed with Imaris 5.7.1 software (Bitplane AG, Zürich, Switzerland).

A relative scale was used to indicate the position of the mouth opening and the genital pores, as defined by Rieger and Sterrer [28]. The total body length equals 100 units (U), whereby the most anterior part of the specimen marks U0 and the most posterior part U100.

## Results

### Landmarks of the life cycle and gross anatomy of *S. roscoffensis*

**Figure 1:**
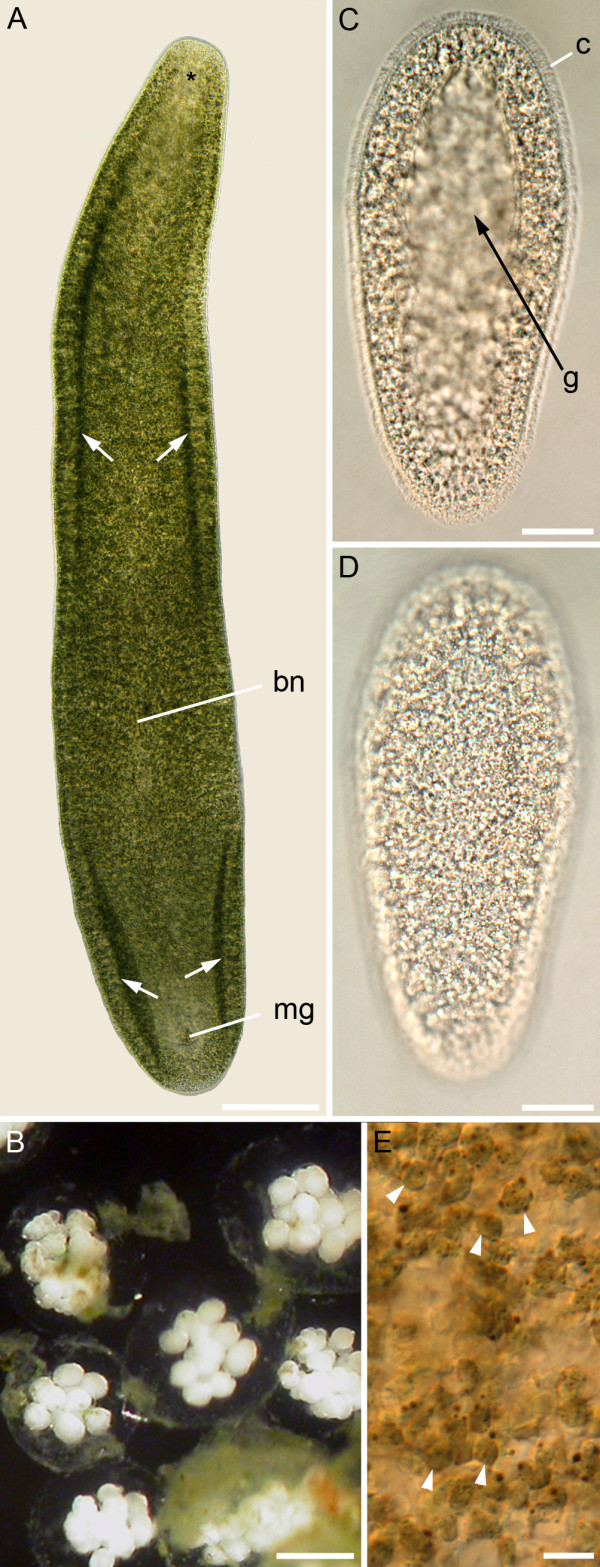
**Developmental stages of *S. roscoffensis*.** A. Green adult in ventral view with anterior facing upwards. The location of the statocyst is marked with an asterisk. Arrows point to the lateral edges, which are looped ventrally to form a ventral groove. The male genital pore (mg) and the bursal nozzle (bn) are located in the posterior half of the animal. B. Up to 20 embryos may be found in a single cocoon. C and D. Newly hatched juveniles yet devoid of symbiotic algae. Ventral view (C), and dorsal view (D), respectively. On the ventral side a groove is formed (g). The animals are completely covered with cilia (c). E. Close-up of the parenchyma of an adult specimen containing algae (arrowheads). Scale bars: 200 μm (A), 100 μm (B), 50 μm (C, D), 20 μm (E).

The adults of *Symsagittifera roscoffensis *are elongated worms with a length of 2–5 mm (Figure 1A). They are dorsoventrally flattened and approximately 350–500 μm wide. The anterior and posterior tip is blunt, the posterior end less than the anterior. The green colour of the adults is due to the presence of the symbiotic algae *Tetraselmis convolutae*. *S. roscoffensis *is a hermaphroditic species with internal fertilization. Within a single cocoon, 10–20 embryos are found (Figure 1B). The embryos show duet-spiral cleavage cf. also [29]. On the fourth day after egg deposition the oval-shaped embryos begin to rotate within the chorion. During the following day individuals hatch from the broken chorion and swim freely in the culture dishes. The white juveniles are about 350 μm long and 140 μm wide (Figure 1C and 1D, additional file 1). As in the adults, the anterior tip is blunter than the posterior tip. Within the next days the white aposymbiotic juveniles take up the free living *Tetraselmis convolutae *from the seawater and gradually acquire the green colour (Figure 1E).

### Myogenesis of *S. roscoffensis*

#### Embryonic musculature

**Figure 2:**
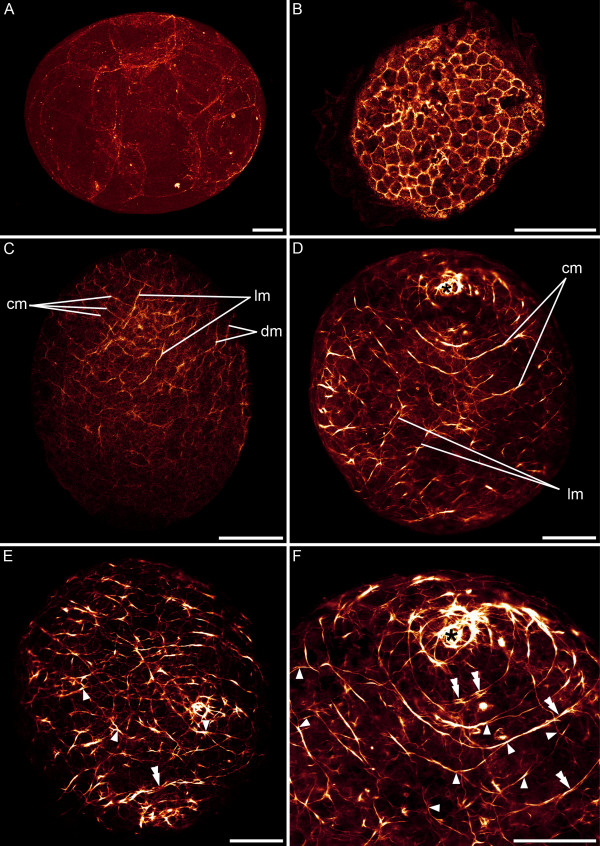
**CLSM micrographs of F-actin labelling in embryonic *S. roscoffensis*.** A+B. Cleavage stages prior to muscle formation. Actin is expressed in the zonulae adhaerentes. A. 5 hours after egg deposition. B. 33 hours after egg deposition. The embryo possesses approximately 370 cells. C. 48 hours after egg deposition. Myogenesis in the anterior pole, facing upwards, precedes the development of muscle fibres in the posterior pole. Circular (cm), longitudinal (lm) and diagonal muscles (dm) are present. D-F. From the third day after egg deposition onwards, the ventral (D) and dorsal (E) sides are distinguishable. The anterior pole (asterisk) is surrounded by a spiral muscle. Additional muscles are formed by branching off from existing muscles (arrowheads). In addition, double-stranded muscles (double arrowheads) are present. F. Ventral anterior half of (D) in higher magnification. Scale bars: 50 μm.

**Figure 3:**
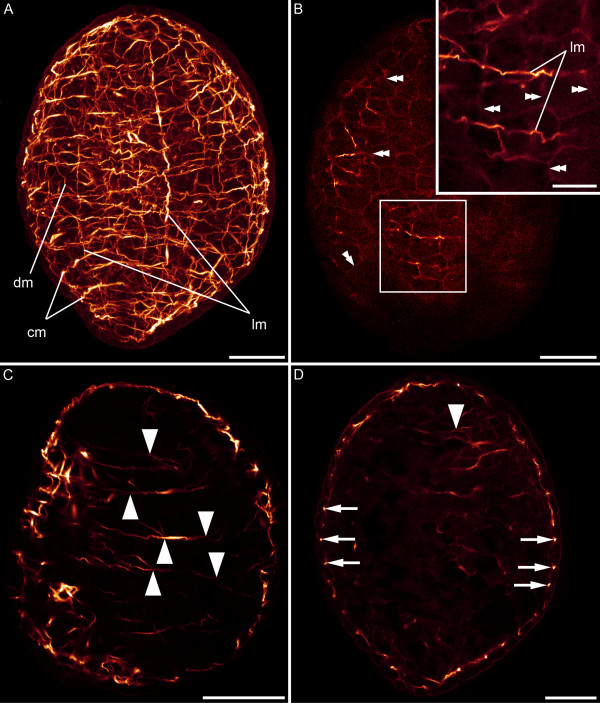
**Embryo on the third day (55 hours, 46% of development between egg deposition and hatching), anterior faces upwards. **A. An orthogonal muscle grid with longitudinal (lm) and circular (cm) muscles is established and very few diagonal muscles (dm) are present. B. Muscles are continuously formed on the basal side of the epidermal cells (double arrowheads point to cell membranes). Inset shows boxed area under higher magnification. Scale bar: 10 μm. C. Muscles emerge by branching off from circular muscles (arrowheads). D. Between cell borders a concentration of actin is visible (arrows). Scale: 25 μm.

**Figure 4:**
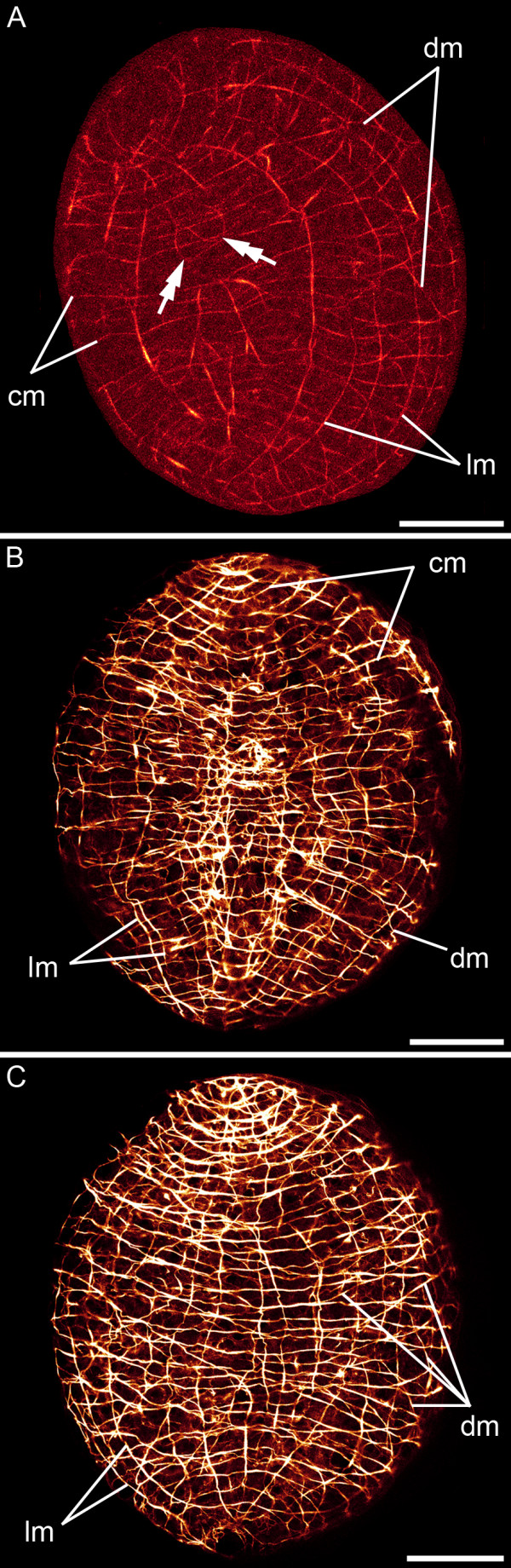
**Embryo on the third day (approx. 65 hours, 54% of development between egg deposition and hatching) with anterior facing upwards. **A. On the ventral side, irregular muscles (double arrows) are visible between the muscle grid of the longitudinal (lm), circular (cm) and diagonal muscles (dm). B. Ventral view. C. Dorsal view. Circular muscles appear denser than longitudinal muscles. Scale bars: 50 μm.

**Figure 5:**
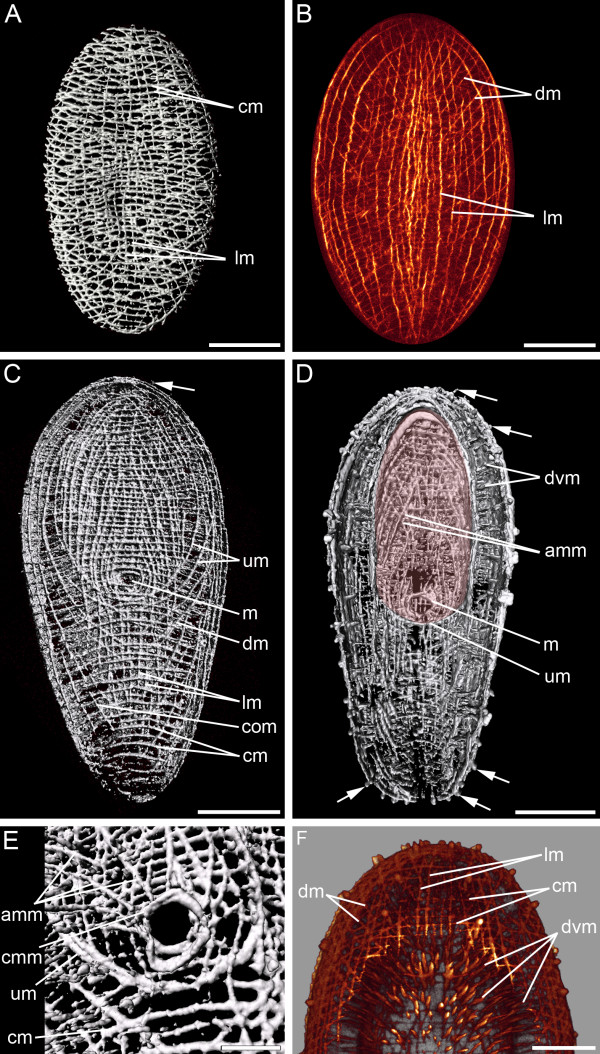
**Musculature of juvenile *S. roscoffensis *shortly before (A, B) and after (C, D) hatching.** All images except B, which is a CLSM micrograph, are 3D reconstructions based on CLSM image stacks. Anterior is up in all aspects. Dorsal and ventral surfaces show different muscle patterns with longitudinal (lm), circular (cm) and diagonal (dm) muscles. A, B. Dorsal views. C, D. The ventral side of the animal contains additional U-shaped muscles (um), ventral crossover muscles (com) and antero-lateral accessory muscles (amm). D. A groove (pink) is formed in the region of the mouth (m). The dorsoventral musculature (dvm) is visible. Filaments of sensory receptor cells are marked with an arrow. E. Close-up of the mouth region, showing the ring muscle around the mouth opening (cmm) and accessory muscles emerging from there. F. Dorsal view of the anterior part showing the complex dorsoventral musculature. Scale bars: 50 μm (A-D), 10 μm (E), 25 μm (F).

In early cleavage stages, the zonulae adhaerentes of cells are stained with phalloidin (Figure 2A). 33 hours after egg deposition the embryo possesses about 370 cells. These cells have a diameter of about 10 μm and a penta- or hexagonal profile. On the third day (40–60% of development between egg deposition and hatching) an orthogonal muscle grid of longitudinal and circular muscles forms along the anterior-posterior axis of the embryo (Figures 2, 3 and 4), and few diagonal muscles become visible (Figure 2C). Short fibres form randomly basally to the epidermis at 40% of development between egg deposition and hatching. The anterior pole is surrounded by a spiral muscle fibre (Figure 2D, F), and the development of muscle fibres in the anterior half precedes myogenesis in the posterior half (Figure 2C–F). The establishment of the muscle grid seems more regular on the future ventral side than on the dorsal side (Figure 2D, E). Solid circular muscles are formed prior to the establishment of complete longitudinal muscles (Figure 2D–F). On the third day (approx. 50 hours and 42% of development between egg deposition and hatching), while a simple muscle grid is already established (Figure 3A), a concentration of actin is exhibited in the borders of surface cells (Figure 3B). Secondary circular muscles form by branching off from already existing circular muscles (Figures 2D–F and 3C) as well as by creating short double-stranded fibre zones, in which new fibres are formed adjacent to existing circular muscles (Figure 2E, F). Throughout the entire embryonic development more circular than longitudinal muscles are present. Accordingly, there are about 30 circular muscles and about 20 longitudinal muscles discernable at the third day after egg deposition (Figure 4). The solid muscle grid of the future ventral side appears more irregular than that of the dorsal side, which is caused by the presence of distinct muscles which probably are associated with the future mouth (Figure 4A and 4B* versus *4C). On the dorsal side, the muscle grid is evenly distributed (Figures 4C and 5A, B). Internal muscles, which are dorso-ventrally orientated, are present at 72 hours (60% of development between egg deposition and hatching). Embryos with an age of about 85 hours (70% of development between egg deposition and hatching) show a ring muscle around the mouth.

#### Musculature of the juvenile

The hatchling possesses longitudinal (approx. 30), circular (approx. 60) and diagonal muscles (Figure 5C–E). The bodywall musculature shows distinct patterns on the dorsal and ventral side, respectively. U-shaped muscles, with the opening pointing towards the anterior side of the animal, are located on the ventral side around the mouth (Figure 5C). On the ventral surface of the animal, a ventral groove leads from the anterior region to the medially positioned mouth (Figure 5D). The mouth has a circular muscle surrounding its opening (Figure 5C, E). Accessory muscles insert at the circular muscle of the mouth and run into antero-lateral direction (Figure 5D, E). On the ventral and dorsal side diagonal crossover muscles extend from the lateral edges of the body and bend towards the midline, but mostly terminate before that (Figure 5). The dorsal diagonal muscles run along the entire body and bend with an angle of approx. 35 degrees relative to the anterior-posterior axis. Posterior to the mouth opening the ventral diagonal muscles bend towards the body midline with an angle of about 55 degrees. Further posteriorly, diagonal muscle fibres curve with an angle of 30 degrees relative to the anterior-posterior axis (Figure 5C). The juvenile already possesses a meshwork of dorsoventral muscles (Figure 5D, F).

F-actin-positive, non-muscular filaments associated with certain sensory cells are present outside the bodywall musculature of juvenile stages still lacking sagittocysts (Figure 5C, D). No specialized musculature in the region of the future genital organs was observed. The number of the distinct bodywall muscles increases with age until the adult stage, while the overall myogenetic arrangement is retained.

#### Adult myoanatomy

**Figure 6:**
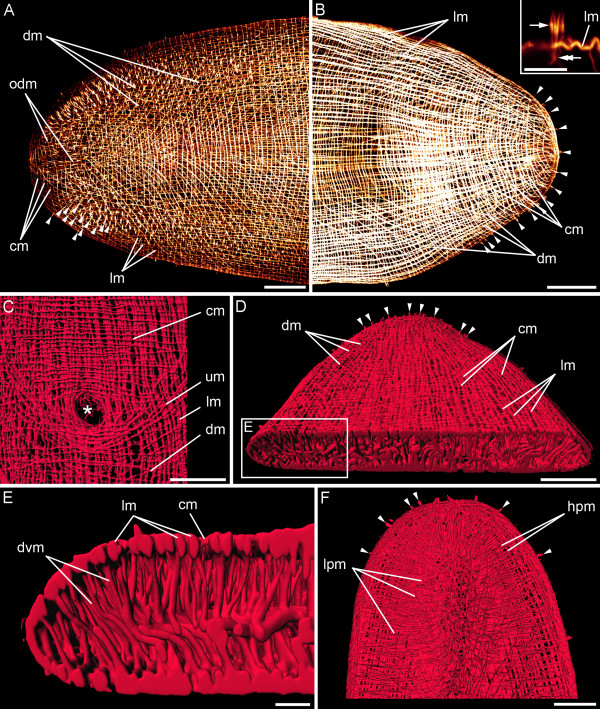
**Adult musculature of *S. roscoffensis*. **A, B. CLSM micrographs. The bodywall contains longitudinal (lm), circular (cm) and diagonal muscles (dm) in the anterior (A) and posterior (B) dorsal part of the animals, respectively. In the anterior part outer diagonal muscles (odm) are present. Arrowheads mark the filaments of sensory receptor cells, which exhibit a collar of microvilli (inset: arrow), three of which being associated with the rootlet (double arrow); scale bar: 5 μm. C-F. 3D reconstructions. C. Close-up of the ventral surface showing musculature associated with the mouth (asterisk). U-shaped muscles (um) curve around the posterior rim of the mouth. D. Dorsal view of the posterior body region. The bodywall musculature encircles the dorsoventral musculature. E. Detail of the boxed area in (D) showing the arrangement of the dorsoventral musculature (dvm). F. The anterior parenchymal musculature is shown by omitting the dorsal bodywall musculature. Horizontal muscle fibres run from the lateral sides to the midline (hpm), while others fan in longitudinal direction (lpm). Scale bars: 50 μm (A-D, F), 10 μm (E).

A network of inner longitudinal and outer circular muscles exists along the entire length of the adult body (Figure 6A, B). Adults have over 300 circular muscles and at least 140 longitudinal muscle fibres. On the outside of the circular muscles outer diagonal muscles are found on the anterior dorsal side of the body. These cross the body midline at an angle of about 65 degrees relative to the anterior-posterior body axis (Figure 6A). Diagonal muscles run from the lateral sides in postero-diagonal direction towards the midline of the dorsal and ventral side, respectively. They cross the body midline at an angle of about 30–50 degrees relative to the anterior-posterior body axis. The longitudinal muscles of the bodywall are positioned underneath all other bodywall muscles (Figure 6A).

*Symsagittifera roscoffensis *exhibits extensive internal (parenchymal) muscles (Figure 6D–F). Apart from the regions close to the genital organs, the statocyst, and in the anterior pole of the animal, dorsoventral muscles connect the ventral and dorsal side of the animal with each other (Figure 6D, E). In the anterior region, parenchymal muscles run from the lateral sides and the anterior tip in horizontal direction to the centre, while others span in an antero-posterior direction and fan in the anterior part of the animal (Figure 6F). The muscles appear to insert at the bodywall musculature (Figure 6D–F). The regular grid of circular and longitudinal muscles is only disrupted by the mouth opening (Figure 6C), as well as by the male and the female genital pore. The mouth is situated at U16 *sensu *[28] of the anterior-posterior body axis. In addition to the sphincter surrounding the mouth, some longitudinal muscles embrace the mouth opening at the lateral sides and separate again posterior to the mouth opening. At the posterior side of the mouth opening some longitudinal muscle fibres emerge and extend in posterior direction (Figure 6C).

**Figure 7:**
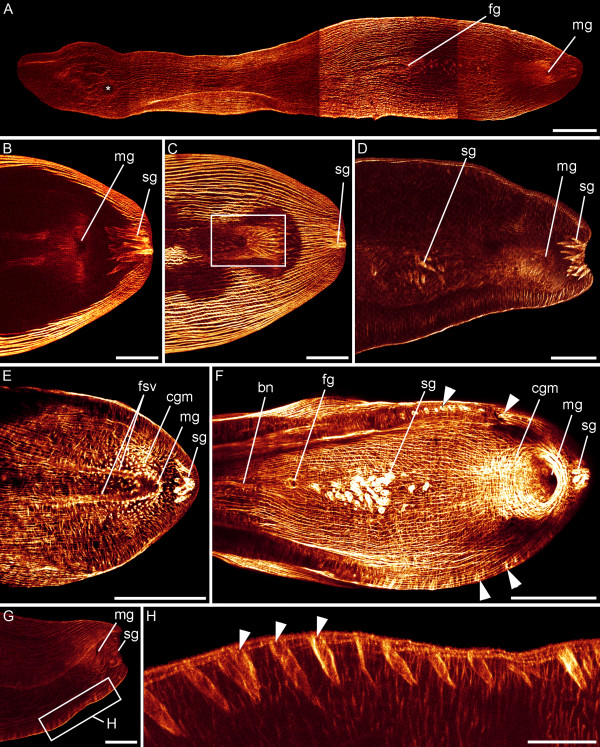
**Adult musculature associated with mouth and genital organs.** CLSM micrographs. Anterior is left in all aspects. A. The relative position of the mouth opening (asterisk) and female (fg) and male gonopore (mg) is visible. B-F. The male gonopore is encircled by circular pore muscles (cgm) and the posterior part of the animal shows sagittocysts (sg). C. The musculature associated with the penis is framed. D. Sagittocysts anterior to the male gonopore are indicated. E. The paired false seminal vesicles (fsv) terminate at the male gonopore. F. The female gonopore and the sclerotized bursal nozzle (bn) are visible. The bursal nozzle leads from the seminal bursa to the female genital opening. Sagittocysts in the lateral sides of the body are marked with arrowheads. G. The muscle mantle of the sagittocysts is located below the bodywall muscle grid. H. Detail of the boxed area in (G), showing the sagittocysts in higher magnification. Scale bars: 200 μm (A, E-F), 50 μm (B, C, H), 100 μm (D, G).

The female genital pore lies in much closer proximity to the male pore than to the mouth, namely at U70 of the anterior-posterior body axis (Figure 7A, F). The bursal nozzle (sometimes also referred to as "bursa mouthpiece"), which is composed of a sclerotized lamellate stack of cells that form a sperm duct, contains intracellular actin (Figure 7F). The male genital pore is situated almost at the posterior end of the body at U90 of the anterior-posterior body axis (Figure 7A–F). The male gonopore is surrounded by circular muscles (Figure 7A, B, E, F). The muscle grid around the male gonopore is not very regular, since many sagittocyst openings are present in this region. The paired "false seminal vesicles", where sperm masses are accumulated, are situated anterior to the male gonopore and are surrounded by parenchymal muscles (Figure 7E).

The muscle mantles of the needle-shaped sagittocysts are situated along the lateral edges and in the posterior region of the body close to the male gonopore (Figure 7F–H). F-actin-positive, non-muscular filaments of microvilli of certain receptor cells are present outside the bodywall along the entire length of the animal (Figure 6). Three of these filaments slightly extend into the body of the animal (Figure 6B, inset).

## Discussion

### Comparison of myogenesis of *Isodiametra pulchra *and *Symsagittifera roscoffensis*

In the two species for which data on myogenesis are available so far, F-actin is only present in the zonulae adhaerentes of the cell walls until 50% (*Isodiametra pulchra*) or 40% (*Symsagittifera roscoffensis*) of development between egg laying and hatching [27], present study. The signal in the zonulae adhaerentes decreases gradually with further development in *I. pulchra *[27]. Such a situation could not be observed in *S. roscoffensis*.

Slightly later, short isolated circular muscles appear in the embryo of *Isodiametra pulchra*, and four or five bands of primary circular muscles encircle the embryo just after 50% of development. The circular muscle bands increase to six to eight muscle bands, followed by single primary longitudinal fibres. These longitudinal fibres emerge independently of each other together with additional circular muscles [27]. In *Symsagittifera roscoffensis*, no such stage in which only circular muscles are present was found. However, we cannot fully exclude the existence of such a stage since such a phase may exist only for a very short ontogenetic period which might have escaped our – albeit tight – developmental analysis.

After establishment of the stomodeum, longitudinal muscles posterior to the mouth bend towards the median anterior-posterior axis in *Symsagittifera roscoffensis *and *Isodiametra pulchra *[27], present study. Circular mouth muscles are visible at stage 7 (88–100% between egg deposition and hatching) in *I. pulchra*, whereas a sphincter around the mouth of *S. roscoffensis *already exists at 70% of development. Already with the presence of the first fibre bands the area of the future mouth shows higher concentration of irregularly arranged muscles than the future dorsal side of *S. roscoffensis*.

Concerning the musculature, differentiation of the dorsal and the ventral side in *Isodiametra pulchra *becomes visible at stage 4 (60–63% of development) [27]. The orthogonal muscle grid is more ordered on the ventral than on the dorsal side in *I. pulchra *[27]. In *Symsagittifera roscoffensis *a dorsal-ventral differentiation of the musculature can be detected on the third day (about 60% of development). The ventral side of *S. roscoffensis *features more irregularly orientated fibres than in *I. pulchra*. The establishment of the regular muscle grid in *S. roscoffensis *seems more unsorted on the dorsal side than on the ventral side. The first diagonal muscles in *I. pulchra *are established at stage 7 (88–100%), whereas in *S. roscoffensis *short diagonal muscle fibres are already present on the third day (42%) [27], present study.

Myogenesis in *Isodiametra pulchra *seems to follow a more regular pattern in the anterior part than in the posterior part of the body [27]. This is not the case in *Symsagittifera roscoffensis*, where the muscle fibres develop simultaneously along the anterior-posterior body axis, but appear more prominent in the anterior pole. In both *S. roscoffensis *and *I. pulchra *the musculature associated with the future sexual organs develops after hatching. After formation of the primary circular muscle fibres, double-stranded secondary circular muscle fibres appear in *Isodiametra pulchra*, similar to the condition found in *Symsagittifera roscoffensis *[27], present study. Secondary muscles arise in both acoels in similar ways: by branching off and by creating double-stranded fibre zones, in which the new muscles form adjacent to primary fibres.

### Comparative analysis of the adult bodywall musculature in Acoela

The general arrangement of the bodywall musculature in *Symsagittifera roscoffensis *resembles that of most Acoela. Longitudinal muscles are positioned underneath the circular and diagonal muscles of the bodywall. Only in *Childiidae *the orthogonal muscle grid consists of outer longitudinal and inner circular muscles [21]. Whereas most of the eight distinct acoel bodywall muscle patterns hitherto described are based on a single or very few species only, the so-called "convolutida"-type comprises seven taxa, namely Actinoposthiidae, Convolutidae, Isodiametridae, Otocelididae, Anaperidae, Sagittiferidae, and Haploposthiidae [12]. These "convolutida"-type acoels together with the Mecynostomidae share the presence of dorsal and ventral diagonal muscles, which both cross the median anterior-posterior body axis [12]. Often, these crossover muscles, which pass immediately behind the mouth, form U-shaped muscles looping around the mouth. Contrary to the "convolutida"-type acoels, the Mecynostomidae possess additional ventral diagonal muscles which are situated anterior to the mouth [12]. The ventral diagonal muscles of *Isodiametra pulchra *are probably not homologous to those of other "convolutida"-type acoels, since the outer dorsal diagonal muscles in *I. pulchra *wrap around the ventral side and form the ventral diagonal muscles, which terminate just in front of the ventral body midline [23,27]. The sagittiferid *Convolutriloba longifissura *lacks ventral diagonal muscles and straight longitudinal muscles anterior to the mouth [24]. Instead, *C. longifissura *possesses roughly concentric fibres around the mouth inside the funnel groove, while these muscles are more eccentric with only the inner-most being concentric in the sagittiferid *Convolutriloba macropyga *[24,26]. Apart from the sphincter muscle,* S. roscoffensis *does not show any concentric muscles around the mouth. At the posterior end of the "convolutida"-type acoels the majority of the straight longitudinal muscles are "special pore muscles" *sensu *[12] that fan from the posterior rim of the mouth and the anterior rim of the gonopore [12]. In *C. longifissura *these muscles cover the entire ventral side of the body, contrary to *S. roscoffensis *and *C. macropyga*, in which most muscles that are positioned posterior the mouth do not fan [24], present study, [26]. As in the sagittiferid species *Antrosagittifera coralline *and *Convolutriloba longifissura*, *S. roscoffensis *exhibits only a few U-shaped longitudinal fibres that flank the mouth [12], present study.

The bodywall musculature of Acoela consists of a grid of longitudinal and circular muscles. The existence of both dorsal and ventral diagonal crossover muscles characterizes all acoel clades except Solenofilomorphidae, Paratomellidae, Diopisthoporidae and some Childiidae [10,21,30]. U-shaped muscles which loop around the posterior region of the mouth are a shared character of acoels and nemertodermatids, but lack in Solenofilomorphidae [10]. By contrast, accessory muscles associated with the mouth opening and parenchymal muscles such as dorsoventral muscles show great overall variety throughout the various acoel clades. Accordingly, a final statement whether or not these muscle sets were part of the ancestral acoel bodyplan cannot yet be made. Comparative analysis of the detailed arrangement of the dorsoventral musculature, however, may yield new character sets for future attempts to reconstruct acoel sistergroup relationships.

### Comparison of acoel inner (parenchymal) muscles

Terms such as "inner", "internal" and "parenchymal" muscles are general expressions for muscles situated inside the acoel bodywall musculature, e.g., dorsoventral muscles, statocyst muscles and copulatory organ muscles. Remarkably few studies have so far focused on the internal muscles, in contrast to the amount of studies which characterize the acoel bodywall musculature. *Childia brachyposthium*, *C. macroposthiu*m and *C. groenlandica *feature highly abundant internal muscles, whereas *C. crassum *shows less numerous internal muscles, and *C. cycloposthium*, *C. submaculatum *and *C. trianguliferum *possess even fewer and weaker internal muscles [21]. The predominant inner muscles in the adult *Isodiametra pulchra *are parenchymal muscles that originate in the region of the statocyst and fan irregularly into posterior and lateral direction [22]. Some fibres reach anteriorly to anchor at the anterior tip of the bodywall musculature [22], while other fibres extend posteriorly into the region of the genital organs [23]. *Praeconvoluta tornuva *shows a dense aggregation of parenchymal muscles in the region of the statocyst, from where fibres extend posteriorly and laterally [31]. Parenchymal muscles were also detected in *Otocelis sp*. and *Mecynostomum sp*., but they are not as prominently developed as in *I. pulchra *[22]. Adult *I. pulchra *exhibit three dorsoventral muscles on each side of the body [22]. In *Mecynostomum sp*. and *Otocelis sp*. the dorsoventral fibres that are associated with the mouth and the ones anterior to it are less developed than in *I. pulchra *[22]. Already at stage 6 (73–87% of development) three pairs of dorsoventral muscles appear in the region of the mouth in *I. pulchra *and become more apparent closer to hatching [27]. *Symsagittifera roscoffensis *possesses dorsoventral muscles almost along the entire anterior-posterior body axis, apart from the region of the statocyst, the genital tracts, and the anterior tip of the animal. As in *I. pulchra*, the inner muscles of *S. roscoffensis *appear to insert either at other inner muscles or at the bodywall musculature [23], present study.

In general, none of the acoel species studied so far exhibits such en elaborated internal musculature, which is best illustrated by its complex arrangement of dorsoventral muscles, as does *Symsagittifera roscoffensis*. However, *S. roscoffensis *lacks internal longitudinal muscles altogether, which in turn are present in, e.g., the mecynostomid species *Paedomecynostomum bruneum *and the isodiametrid species *Pseudoaphanostoma sp*., *Isodiametra pulchra *and *Praeconvoluta tornuva*.

#### Statocyst muscles

Statocyst muscles have been described for many acoel species [17,18]. They were originally thought to be involved in sensory-transduction mechanisms [32], but more recent data suggest that the fibres merely anchor at the statocyst [23]. As a member of the supposedly most basal taxon of the Acoela [33], *Paratomella sp*. exhibits only a few weak parenchymal fibres that anchor in the region of the statocyst [22]. Within the taxon *Childia *the muscles associated with the statocyst are very variable. While only weakly stained statocyst muscles are present in *Childia cycloposthium *and *C. trianguliferum *[21], these muscles, which connect the statocyst to the bodywall, exhibit a star-shaped pattern in *C. crassum *and *C. submaculatum *and a ladder-like grid in *C. brachyposthium *and *C. macroposthium*. *C. groenlandica *displays an intermediate state in which the ventral part of the statocyst shows a star-like arrangement, while the dorsal part is ladder-like shaped. The mecynostomid *Paedomecynostomum bruneum *comprises paired parenchymal muscle strands anchoring at the statocyst, of which one paired muscle extends longitudinally, while the second, shorter paired muscle extends laterally to insert at the lateral bodywall [22]. Similar to the isodiametrid *Otocelis sp*., the mecynostomid *Mecynostomum sp*. only shows a few prominent fibres in the region of the statocyst which reach posteriorly and laterally into the posterior body half [22]. In the isodiametrid *Pseudaphanostoma sp*. parenchymal fibres originate in the anterior region of the statocyst, cross each other, and arch posteriorly to converge at the posterior end of the animal, contrary to the isodiametrid *I. pulchra*, in which the strongest of the fairly short longitudinally orientated statocyst muscles cross each other anterior and posterior to the statocyst [22,23]. Surprisingly, in *Symsagittifera roscoffensis *the extensive internal musculature does not reveal any specific muscles associated with the statocyst. In contrast to that, species with certain statocyst muscles do not show dorsoventral muscles as elaborated as in *S. roscoffensis*. However, the two *Paraphanostoma *species, which lack any specific pattern of statocyst muscles, neither show numerous internal muscles [21]. The arrangement of the musculature associated with the statocyst is very plastic within the various acoel clades, even in closely related taxa such as representatives of Childiidae, and appears therefore unsuitable to resolve higher-taxon relationships.

#### Copulatory organ muscles

Pores in Acoela are by no means simple structures, but have, in general, specific associated muscle systems [34]. The musculature associated with the male copulatory organ is very complex in both *Isodiametra pulchra *and *Symsagittifera roscoffensis *[23], present study. A strong fluorescence signal is also found in the bursal nozzle, which is due to compact sets of actin filaments in the cells that form the tubiform sperm duct in these two species [23], present study. The copulatory musculature of the anaperid *Anaperus singularis *mainly consists of a loose arrangement of muscles surrounding the penis, while the penis of the isodiametrid *Isodiametra earnhardti *possesses outer longitudinal and inner circular muscle fibres [30]. Childiid species possess radial muscles, seminal vesicle ring muscles, "tentacle muscles" *sensu *[21] of the seminal vesicle and inner muscles connecting the base of the stylet with the seminal vesicle wall, whereas in *Childia groenlandica *only a sphincter muscle and inner muscles are present [21,35]. In Sagittiferidae, muscle fibres associated with the male copulatory organs are irregularly arranged, since the male sac-like antrum lacks seminal vesicles [16]. Therefore, the muscles which surround the antrum in *S. roscoffensis *form so-called "false seminal vesicles".

### Acoel sagittocysts and receptor cells

Unique to fifteen acoel species, which are combined in the taxon Sagittiferidae *sensu *[36], sagittocysts are needle-shaped secretory products of sagittocytes, glands which are often surrounded by a muscle mantle around the distal neck [36-38]. Originally, sagittocysts had only been found on the ventral side of the body, particularly in the proximity of the genital pores [15,39-42]. In *Convolutriloba longifissura*, sagittocysts are especially numerous along a sickle-shaped band on the antero-ventral margin and along the posterior margin of the body [37]. In *Symsagittifera roscoffensis*, sagittocysts are mainly found in the region posterior to the male gonopore and along the lateral edges of the animal. Contrary to those of *S. roscoffensis*, the peg-like structures along the lateral edges of *Isodiametra pulchra *are non-muscular filaments of sensory cells, which are located outside the bodywall musculature [43]. Close to the neck of the sagittocyts, fibres of small monociliated sensory receptors are found in *C. longifissura *[37]. The adjacent sensory cell can be considered an integral part of the extrusion apparatus [38]. These sensory cells are devoid of any extensive rootlets and are therefore hard to detect with phalloidin staining, contrary to the so-called "swallow's nest receptors", in which a filament-rich ring of microvilli surrounds the sensory cilia and two or three of these microvilli filaments are associated with the rootlet [43,44]. The sensory cilia are mainly present in the anterior and posterior quarter of the body in *Symsagittifera corsicae *[38]. *S. roscoffensis *and *I. pulchra *exhibit non-muscular filaments of sensory cells in the anterior and posterior region, and in lower number over the entire body surface [43]. No sagittocysts are present in sexually immature *C. longifissura *[37]. Likewise, no muscle mantles of sagittocysts were found in *S. roscoffensis *juveniles. However, non-muscular filaments of sensory receptor cells, which are possibly associated with yet immature sagittocysts, are present at that stage.

The position of the sagittocysts in the posterior tip and close to the male gonopore suggests a role in reproductive behaviour [39,42], while sagittocysts in a rostral position might play a role in prey capture, and those along the dorsal body axis in defence [37].

### Can muscle patterns resolve the internal phylogeny of Acoela?

Although it has recently been shown that morphological characters such as the arrangement of microtubules and axonemes in sperm cells as well as the architecture of the adult musculature are potentially useful characters for phylogenetic analyses [10], only few muscular characters are as of yet available to resolve "convolutida"-muscle type species interrelationships. Based on molecular phylogenetic analysis, the "convolutida"-muscle type taxa Convolutidae, Sagittiferidae, as well as the Haploposthiidae appeared all to be polyphyletic, and the interrelationships of these respective assemblages likewise remained unresolved [10]. Furthermore, 18S rDNA analysis reveals that Convolutidae *sensu *[11], comprising more than one third of all acoel species, cluster in two separate groups [10]. Subsequent morphological analysis revealed that all convolutid species, which possess isodiametric penises with separated longitudinal muscles, cluster separately from the other convolutid species, which instead have penises with longitudinal interwoven or intercrossed muscle fibres [14]. The former assemblage was thus united in the Isodiametridae [14], demonstrating the potential usefulness of features of the muscular bodyplan for phylogenetic inferences.

## Conclusion

Compared to recent data on the myoanatomy of other acoel taxa, *Symsagittifera roscoffensis *shows a highly elaborated myoanatomy. However, additional data especially on the basal taxa Paratomellidae, Solenofilomorphidae and Hofsteniidae are necessary in order to make a final statement about the degree of muscular complexity and the exact muscular ground pattern of Acoela. Nevertheless, the present data available on acoel myogenesis and adult myoanatomy suggest a considerably elaborated musculature with at least longitudinal, circular and U-shaped muscles as being part of the ancient acoel muscular bodyplan. Given the proposed basal position of Acoela within Bilateria, this increases the probability of a relatively complex muscular bodyplan of the last common ancestor of all bilaterian animals.

## Competing interests

The authors declare that they have no competing interests.

## Authors' contributions

AW designed and coordinated research. HS performed research. AW and HS analysed data and wrote the paper. XB provided the study material, contributed data, and commented on the final version of the paper. All authors read and approved the final manuscript.

## Supplementary Material

Additional file 1 Light microscopy scan through a juvenile specimen of *Symsagittifera roscoffensis*, viewed from ventral to dorsal. The light microscopy stack runs through the entire body of the juvenile from the ventral to the dorsal side. Click here for file
